# Mechanostimulation-Induced Cell Adhesion and Interaction with the Extracellular Matrix

**DOI:** 10.3390/biom16010060

**Published:** 2025-12-30

**Authors:** Kazuo Katoh

**Affiliations:** Laboratory of Human Anatomy and Cell Biology, Faculty of Health Sciences, Tsukuba University of Technology, Ibaraki 305-8521, Japan; katoichi@k.tsukuba-tech.ac.jp

**Keywords:** mechanostimulation, signal transduction, cell adhesion, focal adhesion

## Abstract

Cells sense and transmit mechanical forces exerted by their environment to the nucleus via adhesion sites and the cytoskeleton. The nucleus interprets these mechanical inputs and determines cell fate and behavior by regulating gene expression. This review addresses how force-generated signals at the cell–extracellular matrix (ECM) interface influence adhesion, signaling, nuclear function, and tissue remodeling. Disruption of these mechanotransduction pathways contributes to the development of diseases such as cancer, fibrosis, and cardiovascular disorders. Advances in technologies that enable the investigation of the underlying mechanisms will support the development of novel treatment strategies for such diseases.

## 1. Introduction

Cells are not only biochemical entities but also mechanical systems that continuously detect, respond to, and exert physical forces [1]. Mechanical cues originating from the extracellular matrix (ECM), neighboring cells, or intracellular tension are key regulators of cell shape, behavior, and fate [2]. Mechanotransduction, the process by which cells convert mechanical signals into biochemical responses [3], is increasingly recognized as essential not only for normal physiological processes such as development, wound repair, and tissue homeostasis but also for the progression of numerous diseases, including cancer, fibrosis, and cardiovascular disorders [4].

Cell adhesion and interactions with the ECM, a complex three-dimensional network that provides structural and biochemical support for cells, are central to the perception and conversion of mechanical signals. Focal adhesions (FAs) containing integrins sense and transmit forces at the cell–ECM interface, whereas cadherin-containing junctions mediate mechanical coupling between adjacent cells. These forces are propagated through the cytoskeleton to the nucleus, where they influence gene expression by altering nuclear morphology, chromatin organization, and nuclear envelope protein dynamic [5].The nucleus is now understood to be a major mechanoresponsive organelle that integrates force inputs through the linker of nucleoskeleton and cytoskeleton (LINC) complex and related structures [6].

Adhesion to the ECM is essential for tissue development and maintenance, as well as for the development and maintenance of cell morphology and function [7]. ECM protein synthesis, cross-linking, and degradation determine the stiffness of the matrix, which in turn regulates fundamental cellular processes such as migration, growth, and proliferation. Fibroblasts play a key role in the synthesis and secretion of proteins linked to the ECM [8]. The mechanical characteristics of the ECM play a central role in regulating key cellular functions including migration, growth, and proliferation. During locomotion, cells generate traction forces and pull against the ECM, and these forces are sensed at FAs, which transmit mechanical cues to intracellular signaling pathways [9,10,11].

A substantially increased rate of adhesion suggests that mechanotransduction exerts distinct effects on specific cell types and adhesive proteins. Cell proximity to the ECM is an important determinant of adhesion strength, which increases as additional adhesion sites become available. Cell surface receptors interact with ECM components to promote attachment, regulate adhesive potential, and control the spacing between cells as they form adhesions. Correlated assessments of receptor engagement and cell–ECM contact can help clarify how cells monitor and regulate attachment in response to ECM exposure [7].

**Figure 1:**
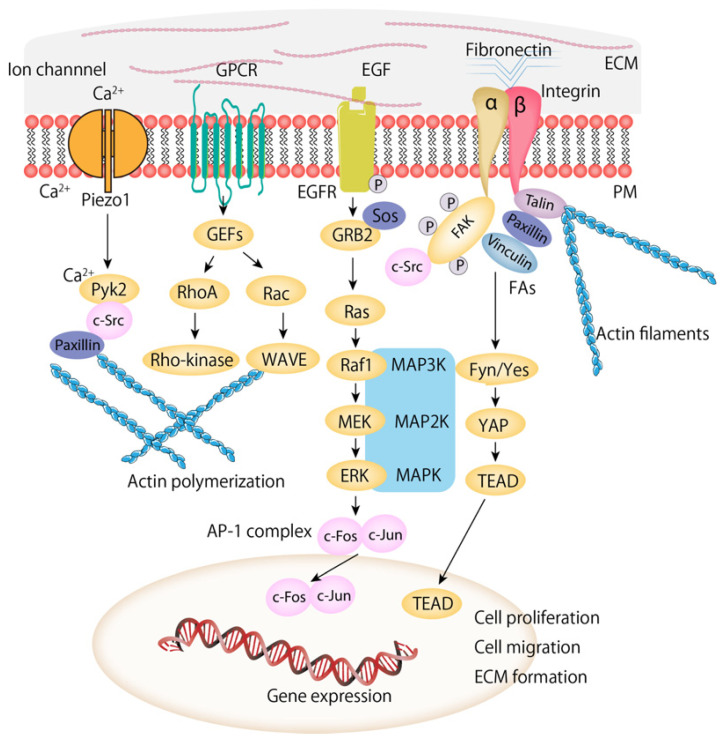
Schematic illustration of mechanotransduction.

The ECM, once regarded as a largely passive scaffold that provided structural support for adherent cells, is now recognized as an active regulator of cell behavior [12]. Its complex three-dimensional architecture is maintained through interactions among numerous protein components [13,14] (Figure 1). Integrins, the primary transmembrane receptors responsible for cell adhesion to the ECM, undergo conformational activation that shifts them from an inactive to an open state. Linker proteins such as talin connect the intracellular domains of integrins to actin filaments, forming the structural basis for FAs and force transmission [8].

Focal adhesions contain integrin sense forces at the cell–ECM interface, whereas junctions that contain cadherin facilitate force transmission between adjacent cells. Mechanical stimuli are transmitted through the cytoskeleton to the nucleus, where they modulate gene expression through changes in nuclear morphology, chromatin structure, and nuclear envelope protein dynamics.

The intracellular pathways mediating mechanical stimuli into the cell include, for example, Piezo1/Pyk2/c-Src/Paxillin, GEF/RhoA/Rho-kinase, GEF/Rac/WAVE, GRB2/RAS/RAF1/MEK/ERK, and FAs/Fyn-Yes/YAP/TEAD. MAPK pathways mediate extracellular information into the cell. MAPK/ERK activation results in phosphorylation of ERK1/2 leading transcription target regulation in the nucleus [15].

The interaction between cells and the surrounding matrix is highly dynamic and bidirectional. Cells exert tension on the ECM, and the matrix in turn resists deformation and remodeling, thereby influencing intracellular cytoskeletal activity [16]. This close biomechanical coupling regulates a wide range of physiological processes, including cell growth, differentiation, and migration [17,18]. A biochemical understanding of mechanotransduction requires detailed knowledge of the variable mechanoresponses of specialized adhesion complexes that anchor cells to the ECM [11].

Accessory proteins such as talin link integrin cytoplasmic tails to actin filaments. Mechanically-induced signaling regulates adhesion stability and activity through the rapid recruitment of mechanoresponsive proteins, including vinculin, paxillin, and focal adhesion kinase (FAK, also known as PTK2), to FA sites in response to mechanical stimulation [13] (Figure 1). Actin filaments extend deeper into the cell and connect to the nucleus through nesprins and other components of the LINC complex, forming a multiprotein continuum that mechanically couples the ECM to the nuclear interior [19].

Through this mechanically continuous protein network, mechanical cues detected at FAs can propagate throughout the cell and ultimately reach the nucleus, where they regulate diverse transcriptional programs, including adhesion-related gene expression, post-translational modifications, and protein conformational changes [11].

The hierarchical integration of mechanical signals across several spatial and temporal dimensions is referred to as multiscale connections. Mechanotransduction functions at three different spatial scales: the micrometer scale of cytoskeletal force transmission and nuclear deformation, the nanometer scale of single integrin molecules undergoing conformational changes upon ligand binding and the millimeter scale of tissue-level ECM remodeling and collective cellular responses. In terms of time, these processes include immediate mechanosensing events from milliseconds to seconds at ion channels and adhesion complexes, intermediate signaling cascades from minutes to hours involving transcription factor translocation and kinase activation and long-term adaptations from days to weeks like epigenetic changes and the development of mechanical memory. It is crucial to comprehend these interrelated scales because disruptions at any level whether tissue-scale alterations in the ECM or molecular mutations impacting protein mechanics [20].

This review discusses the multiscale relationships between mechanical stimulation and cellular responses, with a focus on how mechanostimulation influences cell adhesion, cytoplasmic signaling, nuclear mechanotransduction, and cell–cell communication. We further discuss how dysregulation of these pathways contributes to disease and how recent technological advances have enabled more precise characterization of force-mediated cellular processes. By integrating current knowledge across these interconnected areas, we aim to provide a comprehensive understanding of how mechanical forces shape normal cellular physiology and drive pathological remodeling.

## 2. Mechanical Transduction Process

The cell membrane is the major site for the sensing of mechanical cues from the extracellular environment [21]. Transmembrane proteins specialized for mechanosensing, particularly integrins and cadherins, anchor cells to the ECM and to neighboring cells, respectively [22]. These adhesion molecules function not only as physical connectors but also as mechanosensors that activate signaling cascades in response to mechanical stimulation [23].

Integrins are heterodimeric receptors composed of α and β subunits that bind ECM proteins, such as fibronectin, collagen, and laminin, and connect them to the actin cytoskeleton through adaptor proteins including talin, paxillin, and vinculin [24]. Integrins provide the initial structural link between FA proteins and the ECM and mediate cell adhesion in a tightly coordinated manner [22]. Single-molecule fluorescence resonance energy transfer (FRET) experiments examining integrin structural transitions indicate that ligand binding initiates integrin activation, while talin binding stabilizes the active conformation [25]. Under tension, integrins cluster and recruit cytoplasmic proteins to form FAs, which grow and mature as mechanical load increases [26]. This process strengthens adhesion sites and enhances force transmission [27]. In parallel, FAs activate intracellular signaling pathways, including FAK and Src family kinases, which regulate cell migration, proliferation, and survival [28].

Cadherins, particularly E-cadherin in adherens junctions, mediate homophilic interactions between neighboring cells [29]. These junctions are mechanically reinforced through cytoskeletal attachment proteins such as α-catenin, which undergoes conformational changes under tension to recruit vinculin and strengthen the linkage to the actin cytoskeleton [30]. This mechanical coupling enables tissues to coordinate collective behaviors including epithelial sheet migration and morphogenesis [31].

Mechanosensitive ion channels such as Piezo1 and TRPV4 also contribute to membrane-level mechanosensing [32]. These channels detect membrane deformation or tension and regulate ion flux, which subsequently influences downstream signaling pathways, cytoskeletal organization, and gene expression [33,34,35,36].

Mechanical signals at the membrane interface are strongly dependent on ECM properties, including stiffness, topography, and ligand density [37]. Cells cultured on rigid substrates exhibit enhanced FA formation, increased cytoskeletal tension, and nuclear translocation of mechanosensitive transcription factors such as Yes-associated protein (YAP) and transcriptional co-activator with PDZ-binding motif (TAZ). In contrast, on soft matrices, cells typically display reduced contractility and may enter quiescence or revert to stem-like states [38].

To quantify the forces exerted at individual FAs, techniques such as traction force microscopy and nanopillar imaging have been used to measure how cells apply mechanical load to their substrates [39]. To quantify the forces exerted at individual FAs, techniques such as traction force microscopy and nanopillar imaging have been used to measure how cells apply mechanical load to their substrates [40]. These polypeptide-based force sensors typically consist of an elastic, unstructured peptide linker positioned between fluorophores within a protein domain of known mechanical properties. Changes in FRET between the fluorophores reflect the magnitude of applied force. By inserting these sensors at different positions within mechanically active proteins, it is possible to detect force variations across dynamically coupled molecular complexes [11].

The organization and elasticity of actin filaments, which are modulated by adhesion-dependent pulling forces, can be used to assess a cell’s adhesive response to external mechanical cues such as substrate stiffness, topography, or physiological gradients [41]. Through the transduction of mechanical signals, FAs can activate signaling proteins and initiate downstream signaling cascades [42]. FAs can be visualized by detecting adhesion-related components such as vinculin, or by immunofluorescence labeling of FAK and phosphorylated FAK. Energy-dependent phosphorylation of FAK activates Rho family GTPases, which regulate cytoskeletal dynamics. Cytoskeletal organization is further modulated by proteins such as myosin II, which generates contractile forces by constricting actin filaments [43]. In addition to the cytoskeleton, the cell nucleus plays a central role in distributing mechanical signals throughout the cell via its mechanical connections to the actin network [44].

New approaches for regulating cell behavior increasingly rely on mechanoresponsive elements that induce specific physiological outcomes. Material properties can be manipulated physically, chemically, or biochemically to elicit defined biological responses. Advances in technologies such as force-tunable materials, chemically modified substrates, incorporation of bioactive molecules, nanopatterning, and engineered ECM systems are improving our understanding of cellular mechanotransduction [45].

Protein complexes located at the interfaces between cells and their surrounding matrix mediate extracellular adhesion. FAs, one of the principal classes of these complexes, are composed of integrins that are continuously regulated during adhesion, migration, and cell polarization. Nearby signaling proteins such as FAK modulate cell phenotype by transmitting signals from integrins to downstream pathways. FAK activation through phosphorylation can occur rapidly after the application of mechanical strain or persist during changes in cell adhesion and morphology, including responses to cellular injury [46,47].

**Figure 2:**
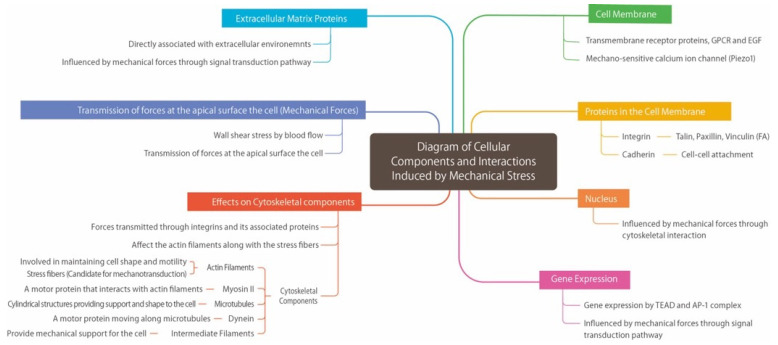
Diagram of cellular interactions through mechanical stress.

Binding of integrin induces the recruitment and activation of downstream FAK signaling components. This leads to stimulation of Ras homolog family member A (RhoA) and its downstream effectors, resulting in actin cytoskeletal reorganization and the formation of membrane protrusions [48,49]. Rho family GTPase pathways and integrin-mediated signaling constitute major routes through which mechanical cues are propagated within cells. Through coordinated signaling along FAK–actin networks, integrin activation promotes cell invasion and cytoskeletal remodeling (Figure 2). Within the Rho pathway, Rac1 drives protrusive activity and regulates glucose uptake, whereas RhoA maintains actin fiber stability and contractility [49].

Electrochemical signaling mediated by mechanosensitive ion channels represents another important pathway of mechanotransduction. In particular, calcium-dependent signaling can rely on mechanically activated ion channels, ATP release, and purinergic receptor activation. This mode of signaling is prominent in neural cells, especially astrocytes, which respond to various forms of mechanical strain through rapid calcium transients and downstream signaling events [50].

Membrane-level mechanosensing initiates a cascade of intracellular events. By the regulated action of adhesion complexes and mechanosensitive channels, cells trigger structural and biochemical responses that determine their behavior and fate. This mechanotransduction level stages the deeper intracellular transmission of mechanical signals to the cytoplasm and nucleus.

Membrane-level mechanosensing initiates a cascade of intracellular events [51]. Through the coordinated activities of adhesion complexes and mechanosensitive ion channels, cells interpret the physical properties of their environment and activate structural and biochemical responses that determine their behavior and fate [52]. These early mechanotransduction processes establish the foundation for the subsequent transmission of mechanical signals into the cytoplasm and ultimately to the nucleus [52].

### 2.1. Diversity of Mechanosensing Mechanisms

Collectively, membrane-level mechanosensing initiates a cascade of intracellular events. Cells decode the physical state of their environment and trigger structural and biochemical responses that define cell behavior and fate through regulated actions at adhesion complexes and mechanosensitive channels. This level of mechanotransduction provides the deeper intracellular transmission of mechanical signals to the cytoplasm and the nucleus.

Besides integrin-based focal adhesions and cadherin-mediated junctions, cells employ an astoundingly diverse array of mechanosensitive receptors and signaling pathways that together enable sophisticated interpretation of the mechanical environment. This allows cells to decode multiple parameters of the mechanical environment that act simultaneously, such as magnitude, duration, frequency, and direction of the applied forces, and thus specify responses in a context-dependent manner.

### 2.2. G-Protein Coupled Receptors as Mechanosensors

Some GPCRs function directly as mechanosensors in addition to their classic functions as biochemical signal transducers. For instance, the angiotensin II type 1 receptor responds to mechanical stretch in a ligand-independent manner through the activation of Gq and G12/13 proteins, with subsequent signaling. This mechanically activated AT1R contributes to stretch-induced hypertrophy in cardiomyocytes and vascular smooth muscle cells. Similar mechanosensitive properties have been described for the bradykinin B2 receptors and endothelin receptors; mechanical forces change the structure of these receptors so that G-protein coupling is favored, even without agonist binding. These reports demonstrate that GPCRs integrate both chemical and mechanical signals and thus enable cells to produce combinatorial responses from simultaneous biochemical and physical cues.

### 2.3. Receptor Tyrosine Kinases and Mechanical Activation

These receptors, including EGFR, VEGFR, and PDGFR, have been demonstrated to be activated not only by their cognate growth factor ligands but also by mechanical forces. Mechanical stretch or substrate stiffness initiates RTK clustering and autophosphorylation, thus activating the downstream signaling cascades that include the ERK/MAPK and PI3K/Akt pathways. This mechanical activation of RTKs points to a mechanism through which physical forces may directly stimulate proliferation and survival pathways in the absence of increased growth factor secretion. In cancer, this mechanical activation of RTK on stiff tumor matrices contributes to ligand-independent growth signaling and therapy resistance [53].

### 2.4. Glycocalyx-Mediated Mechanosensing

The glycocalyx is a dense layer of surface coatings comprised of glycoproteins and proteoglycans. It acts as a mechanosensor, especially in endothelial cells that are subjected to fluid shear stress. Shear forces applied to the glycocalyx are transmitted both to the underlying cortical cytoskeleton and to mechanosensors embedded within it, including integrins and ion channels. Enzymatic degradation of or pathological shedding from the glycocalyx impairs endothelial mechanosensing and contributes to vascular dysfunction in diabetes and inflammation. Thus, the glycocalyx provides a mechanotransduction interface between external fluid forces and cellular responses.

### 2.5. Lipid Bilayer Tension and Membrane Mechanics

The plasma membrane itself serves as a mechanosensor via changes in the tension of the lipid bilayer. Tension in the membrane directly gates mechanosensitive ion channels, including Piezo1/2 and TREK-1, and further modulates the lateral organization of membrane proteins, influencing receptor clustering and signaling complex assembly. Changes in membrane tension also modulate membrane trafficking events, including endocytosis and exocytosis, providing further layers of mechanical regulation. In addition, curvature-sensing membrane proteins, including BAR domain-containing proteins, couple membrane mechanics to cytoskeletal remodeling, thereby allowing the cell to coordinate shape changes with mechanical signaling.

### 2.6. Crosstalk and Integration of Mechanosensing Pathways

Importantly, these various mechanosensing mechanisms do not function in isolation but, rather, demonstrate extensive crosstalk and interconnection. For example, the activation of integrin stimulates GPCR and RTK signaling, while the mechanosensitive ion channel activity modulates focal adhesion dynamics via calcium-dependent signaling. This interconnection is imparted through a common set of downstream effectors—including Rho GTPases, MAPK pathways, and mechanosensitive transcription factors such as YAP/TAZ—acting as nodal points of convergence for several mechanical inputs. The cellular response, therefore, ultimately results from the output integrated by this interwoven mechanosensory network rather than by the action of any single mechanosensor. Appreciation of this diversity and integration is essential to understanding how cells achieve context-specific responses to complex mechanical environments and to understanding how mechanotransduction dysfunction underlies disease [54].

Following detection at the cell membrane, mechanical signals are transmitted through the intracellular architecture, primarily via the cytoskeleton [55]. The cytoskeleton is composed of actin filaments, microtubules, and intermediate filaments, which work together to distribute mechanical forces throughout the cell [56]. These structures function not only as scaffolds for cellular organization but also as active components of mechanotransduction that transmit and integrate physical cues [57].

Actin filaments play a central role in force transmission [58]. Actin stress fibers connect membrane-bound FAs to the perinuclear region, forming a physical continuum between the ECM and the nucleus [59]. Myosin II-mediated contractility generates tension within these fibers, enabling cells to probe mechanical properties of their environment and maintain shape and polarity [60]. This contractile activity is regulated through signaling pathways involving RhoA, Rho-associated kinase (ROCK), and myosin light chain kinase (MLCK), which control actomyosin contractility and maturation of FAs [61].

Microtubules also contribute to mechanical homeostasis and facilitate the intracellular transport of signaling molecules and organelles [60]. They influence FA turnover and communicate mechanical information to the nucleus through motor proteins such as dynein and kinesin [62]. Although microtubules do not transmit force as directly as actin filaments, they are essential for coordinating cellular mechanical responses and integrating signaling across the cytoplasm [61].

Intermediate filaments, including vimentin and keratin, provide structural strength and resistance to compressive forces [63]. They connect cell–cell and cell–ECM junctions and anchor to the nuclear envelope, helping to protect the nucleus from mechanical deformation during stress or migration through confined environments [64].

The LINC complex composed of SUN-domain proteins localized in the inner nuclear membrane and nesprins in the outer membrane is a major structure that mediates the transmission of cytoskeletal forces to the nucleus [65]. These proteins form a physical connection between the cytoskeleton and the nuclear lamina, enabling mechanical forces to act directly on the nucleus and affect chromatin organization and gene expression [66].

Through this integrated system, the cytoskeleton functions as both a transmitter and a modulator of mechanical signals [67]. It combines signals from adhesion complexes, regulates cellular tension, and provides a continuous mechanical linkage along the membrane–cytoplasm–nucleus axis, which establishes a foundation for nuclear mechanotransduction and the regulation of genome activity [68].

Myosin II is a central mechanotransducer that converts biochemical signals into contractile forces, enabling cells to probe and respond to their mechanical environment. This motor protein generates tension within the actin cytoskeleton and governs processes that range from FA maturation to nuclear mechanotransduction [69]. Myosin II exists as a hexameric complex composed of two heavy chains, two essential light chains, and two regulatory light chains. Mammals express three non-muscle myosin II isoforms, NMIIA, NMIIB, and NMIIC, encoded by distinct genes (MYH9, MYH10, and MYH14, respectively), each exhibiting different patterns of tissue-specific expression and specific biochemical properties [70,71]. These isoforms differ in their ATPase activity, the fraction of the mechanochemical cycle spent tightly bound to actin and assembly dynamics, thus enabling the cells to fine-tune contractile responses according to mechanical demands. Myosin II generates force by undergoing ATP-dependent conformational changes that enable it to “walk” along actin filaments. Myosin II-mediated contractility produces intracellular tension along stress fibers that physically link FAs to the perinuclear region during mechanotransduction. Through this actomyosin contractility, cells generate traction forces on the ECM, and the magnitude of these forces correlates with substrate stiffness. High myosin II activity on rigid substrates promotes FA maturation and growth, whereas reduced contractility on compliant matrices results in smaller and more transient adhesions [72].

The regulatory light chain (RLC) of myosin II is primarily phosphorylated at serine 19 and threonine 18. The RhoA–ROCK signaling pathway, in which mechanical stimulation activates RhoA, which then activates ROCK, is the major upstream regulator. This in turn maintains myosin II contractility by directly phosphorylating the RLC and inhibiting myosin light chain phosphatase [73]. MLCK, which responds to calcium–calmodulin signaling, provides an additional calcium-dependent mode of RLC phosphorylation. RhoA activation represents a key convergence point for matrix stiffness, integrin engagement, and growth factor signaling. Mechanosensitive guanine nucleotide exchange factors (GEFs) convert tension into RhoA activation, establishing a positive feedback loop in which increased contractility reinforces further contractile signaling [74].

Tension generated by myosin II supports mechanotransduction across multiple cellular scales. Contractility at FAs induces conformational changes in mechanosensitive proteins such as vinculin and talin, exposing cryptic binding sites that strengthen the coupling of the cytoskeleton to the ECM. This mechanical reinforcement enables force transmission through the membrane to the nucleus via the LINC complex, where nuclear deformation influences chromatin organization and gene expression.

Myosin II contractility also regulates the nuclear localization of mechanosensitive transcription factors, particularly YAP and TAZ. Reduced actomyosin tension results in cytoplasmic retention of these factors, whereas high contractility promotes their nuclear translocation and the transcription of genes associated with proliferation and survival [75].

Dysregulation of myosin II activity contributes to the pathogenesis of multiple diseases. Hyperactivation of the RhoA/Rho-kinase (ROCK)/myosin II axis enhances tumor cell invasion and metastasis in cancer [76,77,78,79].

In fibrosis, persistent myofibroblast contractility establishes a positive mechanical feedback loop that maintains pathological ECM deposition. Thus, targeting myosin II regulation represents a promising therapeutic strategy for conditions driven by aberrant mechanotransduction.

The nucleus is not only the repository of genetic information but also a central mechanoresponsive organelle [80]. Mechanical stress applied at the cell surface is transmitted through the cytoskeleton to the nucleus, where it can directly deform nuclear structure, reorganize chromatin, and regulate gene expression [81,82]. These processes are mediated primarily by the LINC complex, the nuclear lamina, and chromatin architecture [83].

The LINC complex physically connects the cytoskeleton to the nuclear interior. SUN-domain proteins located in the inner nuclear membrane bind to nesprins in the outer membrane, which in turn associate with actin filaments, microtubules, and intermediate filaments [84]. This arrangement allows mechanical forces to be transmitted across the nuclear envelope into the nucleoplasm [67].

The nuclear lamina, composed of A- and B-type lamins, provides mechanical stability and regulates nuclear organization [85]. Lamins interact with chromatin through lamina-associated domains (LADs), influencing gene activation and silencing. Mechanical stress induces phosphorylation of lamin A/C, leading to conformational changes and redistribution throughout the nucleus. These alterations adjust nuclear stiffness and modulate mechanotransduction outcomes [86]. Disruption of lamin structure, as in laminopathies, impairs these processes and contributes to disease [87].

Mechanical stretching of the nucleus can also increase the permeability of nuclear pore complexes (NPCs), which mediate the import and export of transcription factors such as YAP/TAZ and MRTF A [88]. Increased nuclear strain enlarges NPC openings and facilitates transcription factor entry, enhancing gene expression [89]. In contrast, cells on soft substrates or with disrupted LINC complex function exhibit reduced NPC permeability and diminished transcriptional activation [67].

Mechanical forces also reorganize chromatin structure [90]. Physical stress can induce chromatin stretching, histone modification, and repositioning of chromosomal territories [91]. Euchromatin, which is loosely packed and transcriptionally active, can be redistributed toward either the nuclear periphery or interior in response to force, altering accessibility to transcriptional machinery [92]. Force-induced chromatin unfolding facilitates the expression of genes involved in proliferation, differentiation, and stress responses [93].

Recent research has highlighted the roles of nuclear actin and perinuclear actin networks in nuclear mechanotransduction [94]. Nuclear actin dynamics regulate chromatin remodeling, gene expression, and nuclear stiffness [83]. Externally applied forces can induce the formation of perinuclear actin cables that compress and deform the nucleus, altering its mechanical properties and contributing to mechanical memory, a process through which cells retain gene expression patterns elicited by mechanical cues [95].

Taken together, the findings outlined above indicate that nuclear mechanotransduction enables cells to convert mechanical cues into stable transcriptional programs [96]. This process is essential for activities such as stem cell differentiation, cell cycle progression, and tissue remodeling [97]. Dysregulation of nuclear mechanotransduction has increasingly been implicated in disorders including muscular dystrophies, cancer, and premature aging syndromes [98]. Improved understanding of nuclear mechanical properties offers promising opportunities for the development of targeted therapies and advances in regenerative medicine [99].

## 3. Cell Communication

Although most mechanotransduction research has focused on cell–ECM interactions, mechanical communication between cells is equally important, particularly in tissues that require coordinated behavior at the multicellular level [100]. Mechanical forces influence individual cells but can also propagate through cell layers via adherens junctions, tight junctions, and gap junctions, coordinating responses across tissues [101].

Adherens junctions, composed primarily of cadherins, are major mediators of mechanical coupling between neighboring cells [102]. Under tension, E-cadherin interacts with intracellular catenins, especially α-catenin, which undergoes a conformational change that enables vinculin binding [103]. This mechanically induced strengthening enhances linkage to the actin cytoskeleton, allowing tension to be transmitted across multiple cells [104]. Adherens junctions adapt to mechanical load by enlarging and increasing their molecular composition, a process known as junctional remodeling [30].

Tight junctions, although best known for regulating paracellular barrier function, also respond to mechanical forces [105]. Mechanical stress can alter their permeability and influence signaling through ZO proteins and actomyosin contractility, thereby affecting epithelial integrity and polarity [106].

Gap junctions, formed by connexin proteins, permit the direct exchange of ions and small signaling molecules between adjacent cells [107]. Mechanical cues can regulate gap junction assembly, gating, and permeability, impacting calcium signaling, electrical conductance, and synchronized contraction in tissues such as cardiac and smooth muscle [22].

Collective cell migration in development, wound repair, and cancer invasion relies heavily on mechanical communication among cells [108]. Leading-edge cells detect ECM-derived forces and transmit tension through cadherin-based junctions to follower cells, enabling coordinated movement and force generation across the migrating sheet [57].

Disruptions in cell–cell mechanotransduction contribute to epithelial–mesenchymal transition (EMT), barrier breakdown, and tissue disorganization in diseases including cancer and inflammatory disorders [109]. Understanding intercellular force signaling is therefore essential for addressing the complexity of tissue-level mechanics and pathological remodeling [61].

Epithelial tissues maintain structural integrity through specialized junctions that not only provide barrier function but also act as critical mechanosensing hubs. Adherens junctions mediate mechanical coupling through E-cadherin and α-catenin, recruiting vinculin under tension to strengthen cytoskeletal linkages. Tight junctions also respond dynamically to mechanical stress through the modulation of permeability and signaling via ZO proteins and actomyosin contractility [103].

Tricellular junctions, formed where three epithelial cells converge, define a specialized structural domain exposed to a distinct topography of mechanical stresses. Unlike bicellular junctions, tricellular tight junctions (tTJs) contain unique components such as tricellulin, angulin family proteins, and the lipolysis-stimulated lipoprotein receptor (LSR), which assemble into vertical tubular structures that extend along the lateral membrane [110]. Because three cells meet at a single point, tTJs must accommodate geometric constraints and differential tension from multiple directions simultaneously. Recent evidence indicates that tTJs function as sites of mechanical stress concentration, where forces from neighboring cells converge to generate steep local tension gradients that exceed those present at bicellular junctions and may initiate distinct mechanosensitive signaling pathways.

During collective migration and tissue morphogenesis, tTJs undergo continuous remodeling as cells exchange neighbors, requiring rapid mechanical adaptation and coordinated cytoskeletal reorganization [108]. Disruption of tricellular junction integrity compromises epithelial barrier function and contributes to pathological states, although the mechanisms of mechanotransduction at tTJs remain substantially less characterized than at bicellular junctions.

An important but often underappreciated aspect of epithelial mechanobiology is the bidirectional communication between apical junctional complexes and basal integrin-mediated FAs. This vertical axis of force transmission integrates mechanical information from both neighboring cells and the underlying ECM, enabling tissues to coordinate responses across multiple spatial scales [111].

Mechanical tension sensed at adherens junctions is propagated through the cortical actomyosin network to basal stress fibers that terminate at integrin-based FAs [112]. Continuity of force transmission across this network enables cells to coordinate responses throughout tissue layers while simultaneously probing basal matrix rigidity and apical cell–cell contacts. Detection of ECM stiffness by basal integrins establishes a mechanochemical feedback loop that remodels junctional tension and organization through RhoA/ROCK signaling.

YAP and TAZ serve as major integrators of this apical–basal mechanical crosstalk. High tension applied to either basal adhesions or apical junctions promotes YAP/TAZ nuclear translocation, whereas reduced tension at either site results in cytoplasmic retention of these transcriptional regulators [75]. This integrated mechanosensing is evident in the coordinated disassembly of apical junctions and reorganization of basal adhesions that characterize epithelial–mesenchymal transition. Increased integrin engagement with the ECM and reduced tension on E-cadherin act together to activate YAP/TAZ signaling [113,114].

Matrix stiffening in pathological conditions such as fibrosis amplifies basal integrin signaling and alters junctional tension through this coupled mechanotransduction axis, thereby perpetuating disease progression [115]. Elucidating these multiscale mechanical interactions, from tricellular junction stress concentration to apical–basal force integration, is essential for understanding epithelial tissue mechanics in development, homeostasis, and disease.

### 3.1. Fiber Alignment and Contact Guidance

ECM fibrous architecture and cell adhesion dynamics beyond mechanical properties at the bulk scale—such as stiffness—ECM fiber architecture at the micro- and nanoscale importantly controls cell adhesion, mechanosensing, and mechanotransduction. The three-dimensional fibrous structure of the ECM determines fiber orientation, density, diameter, and spacing that presents cells with unique mechanical and topographic signals with strong implications for cellular function [116,117].

Fiber orientation within the ECM dictates the axis of cell polarization, migration, and cytoskeletal force generation due to a process known as “contact guidance”. Cells align preferentially with oriented collagen or fibronectin fibers, elongating along the fiber axis and assembling polarized focal adhesions and stress fibers. The most obvious examples include tissues whose organization follows that of aligned ECM: thus, tendons are composed of tenocytes oriented along parallel collagen fibers running proximal–distal along the axis of mechanical loading [117]. The role of aligned fibers in mechanistically guiding cells is particularly evident during wound healing, where migrating fibroblasts follow the radially oriented fibers to reach the wound center. In cancer, aligned collagen fibers at the tumor–stroma interface are used as “highways” for directed invasion and metastasis. At a molecular level, the basis of contact guidance involves the preferential clustering of integrins along the fiber axis and formation of focal adhesions, where the geometric constraint of the fiber allows for tension development in oriented stress fibers. Evidence from recent studies using nanofabricated substrates demonstrates that even low levels of fiber alignment (10–20° deviation from parallel) induce significant impacts on cell orientation and motility directionality [118].

### 3.2. Effects of Fiber Density and Spacing on Mechanotransduction

Spacing between the ECM fibers determines the size and distribution of adhesion sites available to the cells. Whereas a dense spacing of fibers, such as less than 1 μm, initiates the cell to form many small focal complexes distributed over the cell base, relatively uniform intracellular tension results. On the contrary, when fibers are widely spaced, more than 5 μm, the cell forms fewer but larger, more mature focal adhesions at fiber contact points, generating higher localized traction forces. Such a spacing-dependent adhesion pattern influences downstream mechanotransduction, for instance: sparse fiber networks provoke strong focal adhesion signaling and nuclear YAP/TAZ accumulation, whereas dense networks distribute forces more diffusely, reducing mechanosensitive transcription factor activation [119].

Moreover, migration modes are dependent on fiber density. In dense fibrous networks, cells adapt the mesenchymal mode of migration, which involves integrin-dependent adhesion and proteolytic remodeling of the matrix. In sparse networks or when migrating along individual fibers, cells may use amoeboid migration with reduced adhesion and matrix degradation. Cancer cells exploit this plasticity by switching between modes of migration as a function of ECM architecture to optimize invasion efficiency [120].

### 3.3. Diameter of Fibers and Adhesion Maturation

ECM fiber diameter controls the geometry and maturation state of focal adhesions. Large-diameter fibers (diameter > 500 nm) like collagen I fibrils in physiological tissues allow for large, elongated focal adhesions to nucleate and mature through stepwise recruitment of structural and signaling proteins. Mature adhesions support high traction forces and promote mechanosensitive signaling, including FAK, Src, and ERK pathways. In contrast, small-diameter fibers (diameter < 100 nm) only support small, poorly maturing focal complexes with lower force transmission [121]. Similarly, fiber diameter modulates force transmission mechanics in that thick, rigid fibers resist cellular traction forces, and this permits cells to generate substantial intracellular tension. Thin fibers buckle or deform under cellular forces, offering less mechanical resistance, which reduces intracellular tension generation. This constitutes a mechanical feedback loop in a dynamic, reciprocal cell–matrix mechanical interaction.

### 3.4. Cell-Mediated Remodeling of Fibrous Architecture

Cells actively reshape the fibrous architecture of the ECM through traction forces and proteolytic activity, which in turn creates mechanical feedback loops. Fibroblasts and myofibroblasts exert contractile forces that align, bundle, and compact collagen fibers, thereby stiffening the local ECM and increasing fiber density. Such mechanical remodeling can occur in the absence of changes to ECM composition, revealing that cellular forces are sufficient to dramatically alter matrix mechanics. Meanwhile, cells deposit MMPs that cleave collagen and other ECM proteins, generating local fiber degradation. The balance between fiber compaction and degradation dictates net ECM remodeling outcomes [122,123,124].

In pathologic states, this remodeling process becomes dysregulated. In fibrosis, excessive myofibroblast contractility leads to aberrant collagen alignment and bundling, forming rigid and densely packed fiber networks that perpetuate myofibroblast activation in a mechanotransduction feedback manner, while in tumor microenvironments, cancer-associated fibroblasts reorganize collagen into aligned fiber tracts that are oriented perpendicular to the boundary of the tumor, thereby promoting the invasive protrusion of cancer cells along these remodeled tracts. How the structure of the fibrous ECM modulates cell adhesion and mechanotransduction, and how the cells, in turn, remodel this structure, is essential for understanding proper tissue development, homeostasis, and disease progression.

Changes in ECM stiffness strongly influence cell behavior, including directed migration and cell death following matrix detachment. Substrate stiffness regulates stem cell lineage specification, cell adhesion, motility, and differentiation. For example, soft matrices promote adipogenic differentiation, whereas stiff matrices favor osteogenic differentiation. Cells exhibit preferences for specific stiffness ranges rather than simply migrating toward the stiffest region, and ECM fibers can reorganize and align toward stiffer domains [8].

Mechanical loading not only regulates cellular responses to the environment but also controls how cells remodel the ECM itself, establishing a feedback loop in which cellular activity modifies matrix mechanics, which in turn influences subsequent cellular behavior [64,125].

ECM composition, organization, and stiffness dynamically change in response to mechanical stimuli [126]. A major mechanism underlying this change is the secretion of matrix metalloproteinases, enzymes that degrade ECM components such as collagen and fibronectin, enabling tissue remodeling, cell migration, and invasion [127,128]. Conversely, cells stiffen the ECM by releasing cross-linking enzymes such as lysyl oxidase (LOX), which reinforces collagen fibers and increases matrix rigidity [129].

Mechanosensitive signaling pathways detect changes in ECM stiffness and transduce them into intracellular responses [130]. On stiffer substrates, increased cytoskeletal tension enhances nuclear mechanotransduction and activates transcriptional programs associated with proliferation, differentiation, and matrix synthesis [49]. This feedback is particularly evident in pathological contexts such as fibrosis, where activated fibroblasts deposit excess ECM, further stiffening the matrix and reinforcing their activation in a self-sustaining cycle [6].

ECM remodeling is also influenced by mechanical stretch, shear stress, and compression, which regulate integrin clustering, FA dynamics, and downstream signaling [131]. The physical properties of the ECM, including topography and viscoelasticity, shape cell morphology, polarity, and lineage commitment [4].

The interaction between mechanical forces and ECM remodeling is therefore fundamental to tissue homeostasis, regeneration, and disease progression [132]. Understanding and modulating this bidirectional relationship is essential for developing effective biomaterials and therapeutic strategies in regenerative medicine and oncology [28].

### 3.5. Geometric Constraints and Spatial Limitation in Mechanosensing

Beyond the topics of ECM stiffness and composition, the geometric constraints imposed on cell spreading and shape strongly control mechanotransduction; recent work has shown that “where” a cell adheres is as important as “what” it adheres to. Cell spreading area, aspect ratio, and spatial confinement independently control focal adhesion dynamics, cytoskeletal organization, and mechanosensitive signaling, even when substrate stiffness is held constant [133].

### 3.6. Cell Spreading Area and Mechanotransduction

The total area over which a cell spreads is a critical determinant of the mechanotransduction state of that cell. Micropatterning of adhesive islands of defined geometry has been used to point out the idea that increased cell spreading area progressively activates mechanosensitive pathways: small, confined cells (spreading area < 1000 μm^2^) assemble few, small focal adhesions with low cytoskeletal tension, which in turn promotes cytoplasmic sequestration of YAP/TAZ and diminished proliferation. In contrast, as the area of cell spreading increases (>2000 μm^2^), cells assemble many large focal adhesions and generate higher actomyosin contractility, leading to nuclear YAP/TAZ accumulation and higher proliferation rates.

Importantly, this geometric control operates in a substrate-stiffness-independent manner and overrides it. Indeed, spatially confined cells on stiff substrates exhibit soft-substrate-like behavior, with cytoplasmic YAP/TAZ, while highly spread cells on soft substrates are able to activate YAP/TAZ through increased spreading-induced contractility. This finding uncovers how mechanotransduction integrates multiple mechanical parameters, namely, substrate stiffness, ECM ligand availability, and geometric constraints to specify cellular responses [134,135,136].

### 3.7. Cell Shape and Cytoskeletal Architecture

Cell shape thus directly affects cytoskeletal organization and force transmission. More elongated cells at higher aspect ratios exert prevailing parallel stress fibers oriented along the long axis, channeling traction forces to the poles. More rounded or square cells adopt more radial orientations of the stress fibers and generate forces with a more isotropic pattern. Simultaneously, this shape-dependent cytoskeletal organization dictates nuclear shape and deformation: elongated cells apply higher levels of nuclear strain oriented along the axis of elongation, facilitating mechanotransduction by nuclear envelope stretch and chromatin remodeling [137].

Geometric constraints also play a significant role in the subcellular localization of focal adhesions and signaling complexes. Adhesions preferentially form at cell edges and corners because it is here that the local geometry of cell–ECM contact favors integrin clustering. This kind of spatial patterning of adhesions sets up intracellular gradients of mechanosensitive signals that allow different parts of the cell to maintain discrete mechanotransduction states simultaneously [138].

### 3.8. Implications for Stem Cell Differentiation and Tissue Engineering

This accidental discovery of the fact that geometrical clues regulate mechanotransduction has great consequences for biological research, especially in the field of stem cells and regenerative medicine. Mesenchymal stem cells spread on micropatterned substrates undergo lineage-specific differentiation solely according to cell morphology: spread cells with large adhesion areas preferentially undergo osteogenic differentiation, while rounded, spatially confined cells favor adipogenic differentiation. This geometric control of stem cell fate occurs through the shape-dependent activity of both YAP/TAZ and RhoA/ROCK [139].

For tissue engineering applications, control of cell geometry through scaffold architecture represents a method to instruct cell behavior in the absence of chemical alteration. Micropatterned or nano-topographically defined surfaces allow the control of cell shape and, by inference, alignment and ultimately differentiation in engineered tissues. This consideration points to an important factor in interpreting mechanotransduction studies where any differences in cell spreading area or shape between experimental conditions confound the interpretations based on substrate stiffness. 

### 3.9. Spatial Confinement in Physiological and Pathological Contexts

In vivo, cells are geometrically constrained by surrounding cells and the architecture of the ECM. Compared to isolated cells, which spread on culture substrates, epithelial cells in dense tissues cannot spread as much, which influences their mechanosensitive signaling. During development and morphogenesis, dynamic changes in cell packing density and tissue geometry modulate collective cell behaviors. In pathological contexts, spatial confinement influences cancer cell behaviors: while tumor cells in a dense microenvironment undergo geometric constraints that can suppress cell proliferation through contact inhibition and limitation of cell spreading, cells at the tumor edge or in loose stroma can spread widely, permitting them to promote their proliferation and invasion [140]. Geometric constraints to mechanotransduction involve the integrated understanding of adhesion area, cell shape, substrate mechanics, and the architecture of the ECM. These together define the cellular states for mechanotransduction and require careful control in experimental studies as well as during therapeutic manipulation in regenerative medicine applications.

## 4. Impact of Mechanical Forces on the Physiology of Cells and Mechanotransduction in Disease

Mechanical cues are essential for regulating cellular responses, and impairment of mechanosensory processes can contribute to disease. For example, increasing substrate stiffness enlarges FA sites in fibroblasts, whereas softer matrices produce smaller and more mobile adhesions. FAs form the physical and biochemical link between the ECM and the actin cytoskeleton, demonstrating the intrinsic signaling capacity of the ECM, which enables cells to adapt to mechanical forces [141].

Cells both generate and experience mechanical forces that activate signaling pathways and influence cell fate decisions. Mechanical forces transmitted across tissues regulate processes such as tissue organization, organ development, and the stability of differentiated cell states. Because the nucleus is the largest and stiffest organelle, it is particularly susceptible to deformation and stress, which can reorganize chromosome architecture and alter gene expression programs that drive changes in cellular phenotype [142].

The nucleus is structurally integrated into the cytoskeleton through proteins that span the nuclear membrane, forming physical connections between intranuclear components and cytoplasmic filament networks. These nuclear–cytoplasmic linkages provide mechanical support and positional stability but also transmit forces across the nuclear envelope [143]. By connecting to the cytoskeleton through the LINC complex, the nucleus functions as a mechanosensory structure that enables mechanical inputs to be propagated from the cytoplasm to the genome. Consequently, mechanical signals modulate cytoskeletal–nuclear interactions, providing an additional layer of regulation that influences cellular activity under mechanical stimulation [141,144].

Most research on the influence of force on cell behavior has focused on outside-in mechanotransduction that begins at the cell membrane. Intrinsic membrane proteins, particularly those within focal adhesion complexes, convert mechanical tension into biochemical signals when cells interact with the surrounding ECM. Similar to these outside-in connections, the intracellular cytoskeleton is structurally linked to the nucleus via proteins that span the nuclear envelope. Although these two mechanical pathways differ in structure and signaling mechanisms, both regulate stem cell fate and, when disrupted, contribute to disease [141].

Dysregulation of mechanotransduction is increasingly recognized as a key driver in the onset and progression of numerous diseases [145]. Abnormal sensing or transmission of mechanical stress alters cell behavior, gene expression, and matrix remodeling, producing pathological outcomes [146]. Such disturbances contribute to conditions including laminopathies, cancer, fibrosis, and cardiovascular disorders [147].

### 4.1. Laminopathies

Laminopathies are hereditary disorders caused by mutations in LMNA, which encodes A-type lamins, or in components of the LINC complex such as emerin or nesprins [148]. These mutations compromise the mechanical stability of the nuclear lamina, resulting in nuclei that are abnormally soft, fragile, and prone to rupture under mechanical stress [149]. Faulty force transmission from the cytoskeleton to the nucleus disrupts mechanotransduction, leading to mislocalization of mechanosensitive transcription factors such as YAP and TAZ and to widespread changes in gene expression [150].

These structural and signaling defects are particularly detrimental in mechanically active tissues such as skeletal and cardiac muscle, where they manifest clinically as Emery–Dreifuss muscular dystrophy (EDMD) and dilated cardiomyopathy (DCM) [151]. Affected cells display reduced nuclear stiffness, altered chromatin organization, increased DNA damage, and impaired differentiation, ultimately compromising muscle regeneration and driving progressive tissue degeneration [152].

### 4.2. Cancer, Fibrosis, and Cardiovascular Disease

In cancer, tumor progression is frequently preceded by extensive mechanical remodeling of the tumor microenvironment [153]. Increased ECM stiffness resulting from excessive collagen deposition and cross-linking by LOX enhances integrin signaling and promotes nuclear translocation of YAP and TAZ, driving the transcription of genes involved in proliferation, invasion, and survival [154]. Cancer cells further exploit mechanosensitive pathways to undergo EMT, acquire stem-like properties, and develop resistance to apoptosis [155].

Fibrosis is another pathological consequence of prolonged mechanical stress [156]. Activated fibroblasts, or myofibroblasts, sense matrix stiffening and respond by producing additional ECM, particularly collagen, establishing a positive feedback loop that perpetuates fibrotic remodeling [157]. In organs such as the lung, liver, and heart, progressive fibrosis results in loss of function and irreversible tissue damage [158].

Mechanotransduction also plays a central role in cardiovascular disease. In the vasculature, endothelial cells align and respond according to the magnitude and direction of shear stress generated by blood flow [159,160]. In regions of disturbed flow, mechanosensing is impaired, leading to inflammation, endothelial dysfunction, and the onset of atherosclerosis [161]. Similarly, pathological pressure overload in the heart triggers maladaptive mechanosignaling in cardiomyocytes, promoting cardiac hypertrophy and eventual heart failure [162].

Mechanotransduction defects frequently lie at the intersection of structural failure and dysregulated gene expression [163]. Whether through impaired nuclear mechanics in laminopathies or hyperactivated mechanosignaling in cancer and fibrosis, disruption of mechanical homeostasis destabilizes the balance between force sensing and appropriate cellular response [164]. Understanding these mechanisms is essential for identifying therapeutic targets and advancing mechanotherapeutics, drugs designed to modulate mechanical signaling to restore normal tissue function [165].

### 4.3. Osteoarthritis (OA) and Cartilage Mechanobiology

OA illustrates how altered mechanical loading promotes tissue breakdown due to perturbed mechanotransduction. Normally, chondrocytes of the articular cartilage, under physiological compressive stresses, exert a homeostatic effect on the ECM by balanced synthesis–degradation of proteoglycans and collagen [166,167]. On the other hand, excessive or abnormal mechanical loading due to factors such as joint injury, obesity, or misalignment triggers pathological mechanotransduction cascades.

Under excessive compression, chondrocytes demonstrate increased intracellular calcium signaling through mechanosensitive ion channels, leading to the activation of catabolic pathways by matrix metalloproteinases and aggrecanases. Meanwhile, there is an increase in the production of inflammatory cytokines, participating in a positive feedback manner to enhance cartilage degradation. Altered ECM stiffness in the course of OA further modifies mechanosensing of the chondrocytes: since the loss of proteoglycan-driven softening of the pericellular matrix means the loss of its normal mechanical cues, the cells undergo dedifferentiation and reduced synthetic activity. YAP/TAZ signaling is dysregulated in OA chondrocytes, characterized by changes in nuclear localization that favors hypertrophic differentiation and calcification over the maintenance of healthy cartilage. These insights into the mechanical pathologies provide some therapeutic targets: these include efforts toward the restoration of physiological patterns of loading or pharmacologic modulation of mechanosensitive pathways to prevent the degradation of cartilage.

### 4.4. Pulmonary Diseases and Airway Mechanotransduction

The respiratory system continuously exerts mechanical forces on tissues via cycles of breathing and airflow to critically regulate pulmonary cell behavior and lung homeostasis. Aberrant mechanotransduction is a contributing factor in a range of pulmonary pathologies, such as asthma, COPD, and pulmonary fibrosis [168].

In asthma, airway smooth muscle cells are mechanosensitive to bronchoconstrictor stimuli; in excess, this drives airway remodeling via pathological hypercontractility and increased deposition of ECM and smooth muscle mass due to overactive RhoA/ROCK/myosin II. This process is further amplified through mechanically activated signaling pathways subsequent to cyclic mechanical stretch associated with breathing against a constricted airway. Similarly, in asthma, airway epithelial cells show disrupted mechanotransduction, with disruption of tight junctions and increased permeability occurring under conditions of mechanical stress.

Other mechanotransduction-driven pulmonary diseases include IPF, where progressive stiffening of the lung generates a self-amplifying pathological cycle similar to fibrosis in other organs. Stiff, fibrotic ECM mechanically promotes epithelial–mesenchymal transition in alveolar epithelial cells and differentiation of fibroblasts into hypercontractile myofibroblasts that inappropriately deposit collagen. Ventilator-induced lung injury through excessive stretch-activated mechanotransduction driving inflammatory signaling and barrier dysfunction can also arise as a result of mechanical ventilation in critically ill patients. These examples emphasize the role of mechanical homeostasis in pulmonary health and disease.

### 4.5. Chronic Kidney Disease and Renal Fibrosis

The kidney is an organ that is under constant mechanical stresses brought about by hydrostatic pressure, fluid shear stress, and cyclic stretch imparted by blood flow and filtration. Renal cells, including podocytes in the glomerulus and tubular epithelial cells, possess complex mechanosensing to maintain filtration function and tissue architecture [169].

In CKD, mechanical stress because of hypertension creates a series of maladaptive mechanotransduction cascades. Glomerular podocytes forming the filtration barrier are highly mechanosensitive; high levels of glomerular capillary pressure damage podocytes through over-activation of integrin signaling and eventual cytoskeletal reorganization, resulting in cell detachment, a hallmark of proteinuric kidney diseases. Furthermore, the damaged glomerular basement membrane becomes progressively stiffer and, through this, provides further activation of mechanosensitive pathways to remaining podocytes and mesangial cells.

The common endpoint of CKD progression is tubulointerstitial fibrosis, which clearly exhibits mechanotransduction dysfunction. Tubular epithelial cells subjected to mechanical stretch either by tubular obstruction or increased interstitial pressure undergo partial EMT and secrete pro-fibrotic factors that activate interstitial fibroblasts. These fibroblasts, via integrin-mediated mechanotransduction, perceive the stiffening ECM and differentiate into myofibroblasts, depositing excessive collagen, which self-promotes the fibrotic cycle with positive mechanical feedback. YAP/TAZ signaling also plays a central role in renal fibrosis, where nuclear accumulation of YAP/TAZ in tubular cells and fibroblasts drives pro-fibrotic gene expression programs.

### 4.6. Glaucoma and Ocular Mechanotransduction

Glaucoma is one of the most serious causes of irreversible blindness. It mainly signifies a disease of mechanical stress and failing cellular mechanotransduction at its root. It exposes RGCs and optic nerve head tissues to chronic mechanical strain because of the increased IOP, leading eventually to their death and loss of vision [170,171].

Because RGC axons exiting the eye pass through a collagenous meshwork—the lamina cribrosa—to form the optic nerve, the latter represents a key site of mechanotransduction in glaucoma. The lamina cribrosa undergoes posterior deformation with conditions of elevated IOP, creating focal mechanical stress concentrations that compress and distort passing axons. This mechanical insult provokes axonal transport dysfunction, which leads to RGC degeneration. Astrocytes and other reactive glial cells within the optic nerve head also respond to increased mechanical stress by upregulating the secretion of pro-inflammatory cytokines and reactive oxygen species that amplify the injury to the RGCs. At the level of the individual RGC, mechanical stretch increases calcium influx via mechanosensitive channels, while mitochondrial dysfunction and activation of apoptotic pathways are also activated. Furthermore, anterior chamber trabecular meshwork cells responsible for aqueous humor drainage likewise show mechanosensitive behavior: chronic mechanical stretch leads to ECM remodeling and cellular stiffening that paradoxically reduces drainage capacity in a positive feedback loop that elevates IOP. Recent evidence now indicates that under chronic mechanical stress, the nuclear envelope of RGCs is compromised, and lamin disruption and nuclear membrane rupture are additional causes of RGC death [172]. Understanding such emergent mechanotransduction mechanisms opens up possible therapeutic opportunities beyond traditional IOP-lowering approaches, including neuroprotective approaches targeting mechanical stress responses [171] (see Table 1). 

## 5. Conclusions

Mechanotransduction is the process through which cells interpret and respond to the physical properties of their environment. Mechanical signals detected at adhesion complexes are transmitted through the cytoskeleton and processed within the nucleus through integrated signaling networks that regulate cell shape, function, and fate. Cell–cell communication and ECM remodeling further refine and sustain these mechanical cues, maintaining tissue integrity or driving disease when dysregulated.

This review has illustrated how forces propagate through hierarchical biological systems by systematically pursuing the course of mechanical signal transduction across a number of interrelated scales. At the molecular (nanometer) scale, individual proteins such as talin, vinculin, and α-catenin undergo force-induced conformational changes that expose cryptic binding sites and initiate mechanosensitive signaling cascades. The cytoskeletal networks, composed of actin filaments, microtubules, and intermediate filaments, transfer forces between sites of membrane adhesion and the nucleus via the LINC complex. These molecular events scale up to the cellular (micrometer) scale. Downstream of this, nuclear mechanotransduction orchestrates physical deformation into transcriptional programs via chromatin remodeling, nuclear pore complex regulation, and the import of mechanosensitive transcription factors. Cells then work together over the tissue scale (millimeters to centimeters) to mechanically remodel the ECM through secreted proteases and crosslinking enzyme activity. This sets up mechanical feedback loops that impinge on neighboring cells and perpetuate either pathological progression or homeostatic maintenance of the tissue. Importantly, dysfunction at any one scale propagates through this integrated network: membrane mechanosensing defects in cancer disrupt normal force-regulated behaviors, matrix stiffening at the tissue scale in fibrosis hyperactivates cell contractility, and lamin mutations at the nanoscale in laminopathies compromise nuclear mechanical stability. In the future, therapeutic approaches aimed at mechanotransduction will need to consider this process as one that intrinsically spans multiple scales, targeting the integrated mechanical circuitry that exists from molecules to tissues rather than its individual parts.

This review highlighted the central role of nuclear mechanotransduction in controlling gene regulation and cellular identity. A deeper understanding of mechanically activated cell–ECM interactions offers promising opportunities for therapeutic intervention, regenerative medicine, and the design of biomaterials that harness mechanical cues to guide controlled cellular behavior.

## Figures and Tables

**Table 1:** Mechanotransduction across cellular compartments and its implication in disease.

Compartment	Mechanosensitive Structures	Mechanical Inputs	Mechanotransduction Events	Relevance in Disease	References
Extracellular Matrix (ECM)	Collagen, fibronectin, laminin, proteoglycans, LOX, MMPs	Matrix stiffness, topography, strain	ECM composition and organization dictate ligand availability and mechanical resistance.	Stiffened ECM is characteristic of tumors and fibrotic tissue.	[35,164]
Cell Membrane	Integrins (α/β subunits), Piezo1/2, TRPV4, caveolae, 108s	Shear stress, compression, cyclic stretch	Integrins cluster under force, forming focal adhesions linked to actin cytoskeleton.	Shear-induced activation of Piezo1 is critical in endothelial cell function.	[33,34,35]
Cytoskeleton	Actin stress fibers, myosin II, microtubules, intermediate filaments	Tension, compression, topographical cues	Actomyosin contractility generates intracellular tension.	RhoA/ROCK signaling is upregulated in many cancers.	[77,78,79]
Nuclear Envelope & Lamina	LINC complex (SUN1/2 + nesprins), Lamin A/C, emerin	Cytoskeletal traction, matrix stiffness	Forces are transmitted via the LINC complex to deform the nucleus.	Lamin mutations disrupt nuclear stiffness causing muscular dystrophies and premature aging syndromes.	[144]
Chromatin & Gene Regulation	Chromatin (euchromatin/heterochromatin), histones, transcription factors	Nuclear deformation, mechanical memory	Stretch-induced chromatin decondensation alters gene accessibility.	Aberrant YAP/TAZ activation promotes tumor growth and EMT.	[134,135]
Cell–Cell Junctions	E-cadherin, α/β-catenin, vinculin, gap junctions (connexins)	Intercellular force transmission, epithelial tension	E-cadherin mediates adherens junctions; under tension, α-catenin recruits’ vinculin to strengthen linkage to actin.	Reduced E-cadherin tension sensing facilitates EMT and invasion.	[113,114]

## Data Availability

Not applicable.

## Abbreviations

The following abbreviations are used in this manuscript:

DCM	dilated cardiomyopathy
ECM	extracellular matrix
EDMD	Emery–Dreifuss muscular dystrophy
EMT	epithelial–mesenchymal transition
FA	focal adhesion
FAK	focal adhesion kinase
FRET	fluorescence resonance energy transfer
GEF	guanine nucleotide exchange factor
GPCR	G-Protein Coupled Receptors
LAD	lamina-associated domain
LINC	linker of nucleoskeleton and cytoskeleton
LOX	lysyl oxidase
LSR	lipolysis-stimulated lipoprotein receptor
MLCK	myosin light chain kinase
MMPs	matrix metalloproteinases
NPCs	nuclear pore complexes
OA	Osteoarthritis
RhoA	Ras homolog family member A
RLC	regulatory light chain
ROCK	Rho-associated kinase
TAZ	transcriptional co-activator with PDZ-binding motif
tTJ	tricellular tight junction
YAP	Yes-associated protein
